# Preterm and Early-Term Delivery After Heat Waves in 50 US Metropolitan Areas

**DOI:** 10.1001/jamanetworkopen.2024.12055

**Published:** 2024-05-24

**Authors:** Lyndsey A. Darrow, Mengjiao Huang, Joshua L. Warren, Matthew J. Strickland, Heather A. Holmes, Andrew J. Newman, Howard H. Chang

**Affiliations:** 1 Department of Epidemiology, Biostatistics, and Environmental Health, School of Public Health, University of Nevada, Reno; 2 Geriatric Research and Education Clinical Center, Veterans Affairs Health Care Systems, Palo Alto, California; 3 Department of Biostatistics, School of Public Health, Yale University, New Haven, Connecticut; 4 Department of Chemical Engineering, John and Marcia Price College of Engineering, University of Utah, Salt Lake City; 5 Research Applications Laboratory, National Center for Atmospheric Research, Boulder, Colorado; 6 Department of Biostatistics, Rollins School of Public Health, Emory University, Atlanta, Georgia

## Abstract

**Importance:**

Heat waves are increasing in frequency, intensity, and duration and may be acutely associated with pregnancy outcomes.

**Objective:**

To examine changes in daily rates of preterm and early-term birth after heat waves in a 25-year nationwide study.

**Design, Setting, and Participants:**

This cohort study of singleton births used birth records from 1993 to 2017 from the 50 most populous US metropolitan statistical areas (MSAs). The study included 53 million births, covering 52.8% of US births over the period. Data were analyzed between October 2022 and March 2023 at the National Center for Health Statistics.

**Exposures:**

Daily temperature data from Daymet at 1-km^2^ resolution were averaged over each MSA using population weighting. Heat waves were defined in the 4 days (lag, 0-3 days) or 7 days (lag, 0-6 days) preceding birth.

**Main Outcomes and Measures:**

Daily counts of preterm birth (28 to <37 weeks), early-term birth (37 to <39 weeks), and ongoing pregnancies in each gestational week on each day were enumerated in each MSA. Rate ratios for heat wave metrics were obtained from time-series models restricted to the warm season (May to September) adjusting for MSA, year, day of season, and day of week, and offset by pregnancies at risk.

**Results:**

There were 53 154 816 eligible births in the 50 MSAs from 1993 to 2017; 2 153 609 preterm births and 5 795 313 early-term births occurring in the warm season were analyzed. A total of 30.0% of mothers were younger than 25 years, 53.8% were 25 to 34 years, and 16.3% were 35 years or older. Heat waves were positively associated with daily rates of preterm and early-term births, showing a dose-response association with heat wave duration and temperatures and stronger associations in the more acute 4-day window. After 4 consecutive days of mean temperatures exceeding the local 97.5th percentile, the rate ratio for preterm birth was 1.02 (95% CI, 1.00-1.03), and the rate ratio for early-term birth was 1.01 (95% CI, 1.01-1.02). For the same exposure, among those who were 29 years of age or younger, had a high school education or less, and belonged to a racial or ethnic minority group, the rate ratios were 1.04 (95% CI, 1.02-1.06) for preterm birth and 1.03 (95% CI, 1.02-1.05) for early-term birth. Results were robust to alternative heat wave definitions, excluding medically induced deliveries, and alternative statistical model specifications.

**Conclusions and Relevance:**

In this cohort study, preterm and early-term birth rates increased after heat waves, particularly among socioeconomically disadvantaged subgroups. Extreme heat events have implications for perinatal health.

## Key Points

**Question:**

Are heat waves associated with increased rates of preterm birth and early-term birth in the US?

**Findings:**

In this cohort study of 53 million births, daily rates of preterm and early-term births were positively associated with heat waves, with stronger associations observed after heat waves of longer duration and higher mean temperatures.

**Meaning:**

This study suggests that the increasing frequency and intensity of heat waves have implications for perinatal health in the US.

## Teaser

This cohort study examines changes in daily rates of preterm and early-term births after heat waves over a 25-year period in the US.

## Introduction

Preterm birth (delivery at <37 weeks’ gestation) is a leading cause of infant mortality and longer-term morbidities, including respiratory, cognitive, and behavioral outcomes.^1^ Early-term infants (delivery at 37-38 weeks’ gestation) also experience increased morbidity and mortality relative to those born at 39 to 40 weeks’ gestation.^2,3,4^ In the US, these outcomes affect many infants, with 10.5% of all infants born preterm and 28.8% born early term.^5^ Given the high baseline risks and the influence on lifetime health trajectories of preterm and early-term births, factors that increase these pregnancy outcomes even modestly have large public health implications.

Previous research suggests an acute association of high outdoor temperatures in the week preceding birth with preterm birth.^6,7^ Studying heat waves is considerably more difficult because of the large sample sizes required to estimate modest effects of rare multiday heat events. With a few exceptions, existing studies generally support a positive association between heat waves and preterm birth, but with considerable heterogeneity in the magnitude of effect estimates observed, the definition of heat waves, and the heat thresholds.^8,9,10,11,12,13,14,15,16^ Although few studies directly address physiological mechanisms, there are several biologically plausible pathways. Heat stress and dehydration reduce uterine and placental blood flow, possibly affecting uterine contractility and/or hormone levels governing the induction of labor.^17,18^ Heat stress causes oxidative stress and the release of heat shock proteins that may trigger an inflammatory cascade, plausibly affecting the induction of labor.^18,19^ Extreme heat may also trigger the premature rupture of membranes,^20^ leading to labor (spontaneous or medically induced); alternatively, it may motivate a medically indicated delivery by exacerbating maternal morbidities (eg, hypertension) or fetal distress.

In this study, we estimated the changes in rates of preterm and early-term births in response to heat waves, defined as multiday periods of extremely high temperatures relative to local norms. We investigated these associations using birth record data from the 50 largest US metropolitan areas, encompassing both urban and suburban areas, over a 25-year period to capture numerous heat wave events of variable duration and intensity. We applied previously validated methods that account for seasonally varying risk sets of ongoing pregnancies in each location. The results provide nationally representative and precise population-level associations for an exposure that has been historically rare but is increasing due to global climate change.^21^

## Methods

The project was approved by the University of Nevada, Reno institutional review board. The informed consent requirement was waived due to minimal risk to participants and because the research could not be practically carried out without the waiver. This report followed the Strengthening the Reporting of Observational Studies in Epidemiology (STROBE) reporting guideline for observational studies.

### Study Population

Natality data in the US for the birth years from 1993 to 2017 were obtained from the National Vital Statistics System at the National Center for Health Statistics (NCHS), Centers for Disease Control and Prevention. State and county Federal Information Processing Standards codes were used to identify births to mothers residing in the 50 largest metropolitan statistical areas (MSAs), based on the MSA-county delineation and populations in the 2010 US Census (416 counties total). Names and counties of MSAs are shown eTable 1 in Supplement 1. The 2 largest MSAs (Los Angeles–Long Beach–Santa Ana, California; and New York–Northern New Jersey–Long Island) were further subdivided.^9^ For simplicity, these 53 locations (48 MSAs and 5 sub-MSAs) are henceforth referred to as *MSAs*. The MSA was selected as the spatial scale of analysis to achieve high numbers of daily birth counts in each location while also limiting the geographic area assigned to shared heat wave exposure. Date of birth, state, county, gestational age, maternal age, maternal educational level, maternal race and ethnicity, infant sex, parity, induction of labor, and plurality were extracted from birth files and harmonized to accommodate birth record field changes over time. Race and ethnicity were maternal-reported on the birth record as Hispanic or non-Hispanic ethnicity and Alaska Native, American Indian, Asian, Black, Other Pacific Islander, or White race. To achieve sufficient numbers for analysis categories, these groups were collapsed into Hispanic, non-Hispanic Black, non-Hispanic White, and non-Hispanic other race. To examine possible effect modification of the association between heat waves and birth outcome by race and ethnicity and other factors, data were stratified by maternal age group (<25, 25-34, and ≥35 years), maternal educational level (≤high school, General Educational Development certification, or 12 years of school; associate’s or bachelor’s degree or 13-16 years of school; advanced degree or master’s, doctoral, or professional degree or ≥17 years of school), maternal race and ethnicity (self-reported then broadly categorized into any Hispanic origin; non-Hispanic Black; non-Hispanic White; or non-Hispanic other race, including American Indian, Alaska Native, Asian, Other Pacific Islander, and unknown), parity (eg, first born, second born), and infant sex. Preterm birth was defined as birth between 28 weeks and 0 days’ gestation and 36 weeks and 6 days’ gestation, and early-term birth was defined as birth between 37 weeks and 0 days’ gestation and 38 weeks and 6 days’ gestation. Extreme preterm births (≤27 weeks; 6% of preterm births) were excluded a priori due to greater association with intrauterine infection and congenital anomalies and to be consistent with our previous analysis.^1,9,22,23^

### Meteorological Data

Daily minimum and maximum temperatures were obtained at 1 × 1-km resolution for the continental US from Daymet, a National Aeronautics and Space Administration–supported product from the Earth Science Data and Information System and the Terrestrial Ecology Program.^24^ Mean daily temperatures (mean of Daymet minimum and maximum) were spatially averaged over grids in each county, and then county-level estimates were combined into a single population-weighted spatial mean for each MSA on each day. County population weighting was based on the 2010 US Census population weights to maintain consistency over time.

### Heat Waves

Hot days in each MSA were defined as those exceeding the 97.5th percentile threshold of the MSA-specific temperature distribution over the 25-year study period. Identification of heat waves was based on clusters of hot days within 2 acute exposure windows: the 7 days (lag, 0-6 days) and 4 days (lag, 0-3 days) preceding the birthdate, selected based on previous findings of positive associations in the 7-day window,^9^ and evidence for heat stress and other outcomes suggesting a shorter lag time.^25^ Heat waves were defined 3 ways to capture aspects of duration and intensity: (1) heat wave definition 1 (HW1): total number of hot days in the 4-day (or 7-day) window, represented by an ordinal variable with categories of 0, 1, 2, 3, or 4 days or more; (2) heat wave definition 2 (HW2): consecutive hot days in the 4-day (or 7-day) window, represented by binary indicators for 2 or more consecutive days, 3 or more consecutive days, or 4 or more consecutive days; and (3) heat wave definition 3 (HW3): mean degrees Celsius over the threshold during the exposure window, a continuous variable calculated as the 4-day (or 7-day) moving mean − the 97.5% threshold, and if less than 0, then set to 0.

### Statistical Analysis

Data were analyzed between October 2022 and March 2023 at the NCHS. A methodological challenge in studies of heat waves (or temperature) and pregnancy duration is the well-documented seasonal variation in conceptions^26,27^ and the corresponding peak of pregnancies nearing term each year in July in the US when hot temperatures also peak, creating potential for confounding.^28^ Our analytic approach was designed to avoid this bias and was informed by a simulation study evaluating different statistical approaches^29^ that supported the use of a time-series approach incorporating control for the risk set of ongoing pregnancies and their gestational age distribution,^30^ because the risk of preterm or early-term birth increases sharply as a pregnancy nears term. For each day in each MSA, counts of preterm and early-term births were tabulated, and pregnancies at risk during each gestational week enumerated. Daily outcome counts were then linked to the exposure metrics indicating the occurrence of heat waves in the previous 4 (or 7) days. Overdispersed Poisson regression models with scaled (Pearson) SEs included an offset term representing the expected count of preterm (or early-term) births on each day based on the counts and gestational ages of ongoing pregnancies. More details of the offset and model specifications are available in the eMethods in Supplement 1.

Analyses were restricted to the warm season (May to September), and models included control for location (52 indicators), day of week (6 indicators), birth year (24 indicators), and a cubic spline on day of warm season (May 1 = 1 and September 30 = 153) to adjust for recurrent seasonal trends (1 knot on July 15, 4 total *df*). The last day included in preterm birth analyses was August 17, 2017, because full enumeration of pregnancies at risk beyond that date would require birth records from 2018.

Sensitivity analyses were conducted to examine the robustness of results to different assumptions. Heat waves were redefined by (1) applying a 95% temperature threshold to define a hot day and (2) minimum and maximum daily temperature metrics from Daymet instead of the mean (97.5% threshold). In another analysis, medically induced births were excluded from daily counts of preterm and early-term births (but appropriately retained in daily risk sets). In addition, instead of conducting 1 overall model controlling for MSA, we conducted 53 MSA-specific models, allowing the day of season, birth year, and weekday covariate control to be MSA specific. The 53 risk ratios for each heat wave metric were then combined in a random-effects meta-analysis; details of this model are provided in the eMethods in Supplement 1.

With the exception of the Daymet temperature data processing, all analyses were conducted onsite at the NCHS Research Data Center (Centers for Disease Control and Prevention) due to the restricted-use birthdate and geography variables and the central importance of these fields to the study questions. Analyses were conducted using SAS, version 9.4 (SAS Institute Inc) and R, version 4.2.1 (R Project for Statistical Computing). Calculated *P* values were from 2-sided tests.

## Results

**Figure 1.**
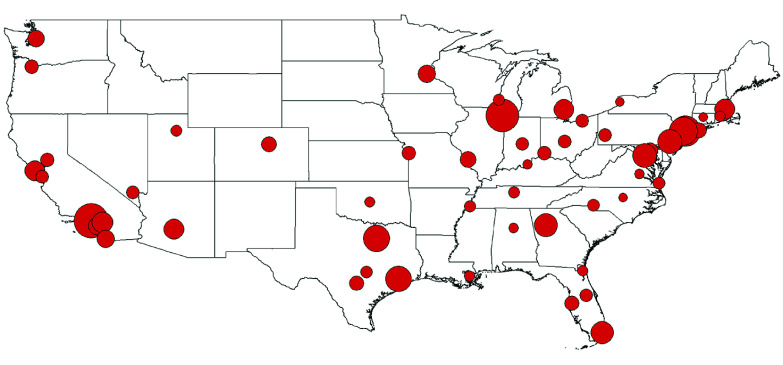
Metropolitan Statistical Areas Included in the Study Circles are proportional to sample size.

A total of 55 748 869 births occurred in the included MSAs between 1993 and 2017, covering 52.8% of all US births during the time period. eTable 2 in Supplement 1 presents the total births by MSA, as well as the counts of preterm and early-term births analyzed after exclusions and warm season restriction. There were 53 154 816 births available after exclusions for multiple births (n = 1 889 189), missing gestational age (n = 381 458), and gestational age of 27 weeks or less (n = 323 406). A total of 30.0% of mothers were younger than 25 years, 53.8% were 25 to 34 years, and 16.3% were 35 years or older. Self-reported maternal race and ethnicity on the birth record indicated 26.1% Hispanic, 17.2% non-Hispanic Black, 48.8% non-Hispanic White, and 7.9% non-Hispanic other race. Figure 1 shows the study locations and relative birth sample sizes. In total, 2 153 609 preterm births and 5 795 313 early-term births occurring during the warm season were analyzed.

The Table shows the number of days in the analysis meeting each heat wave definition for the 4-day (lag, 0-3 days) and 7-day (lag, 0-6 days) exposure windows. For example, there was a mean (SD) of 2.0 (0.7) days per year per MSA that met the definition of a 4–consecutive day heat wave in the 4-day exposure window, and a mean (SD) of 4.2 (0.9) days per year per MSA met the definition of a 4–consecutive day heat wave in the 7-day exposure window. Heat waves were more common in later years of the study. For example, between 1993 and 2004, 1.8 days per year per MSA met the above definition, whereas 2.4 days per year per MSA met the definition from 2005 to 2017. Compared with the 4-day exposure window, more days indicated heat waves in the 7-day window for HW1 and HW2, because the longer window provided more opportunity for a multiday heat event to occur, but fewer days were above 0 for HW3, because a 7-day mean above the 97.5th percentile is more extreme than a 4-day mean above the same threshold.

**Table.** Number of Days in Analysis Meeting Each HW Definition in the 4 Days (Lag, 0-3 Days) or 7 Days (Lag, 0-6 Days) Preceding Birth

HW definition	4-d Exposure window			7-d Exposure window		
	Total No. (%) of days^a^	Mean (SD) No. of days per year and MSA^b^	Range^c^	Total No. (%) of days^a^	Mean (SD) No. of days per year and MSA^b^	Range^c^
HW1^d^						
1 d	11 228 (5.5)	8.5 (1.5)	4.7-11.2	13 225 (6.5)	10.0 (2.1)	5.4-13.7
2 d	7164 (3.5)	5.4 (0.9)	3.1-7.4	9297 (4.6)	7.0 (1.9)	3.6-11.4
3 d	3821 (1.9)	2.9 (0.4)	2.0-3.6	5569 (2.8)	4.2 (0.7)	2.3-5.3
≥4 d	2602 (1.3)	2.0 (0.7)	0.8-4.2	7062 (3.5)	5.3 (1.3)	1.3-7.9
HW2^e^						
≥2 d	12 913 (6.4)	9.7 (0.5)	8.4-10.8	20 640 (10.2)	15.6 (1.1)	13-18
≥3 d	5860 (2.9)	4.4 (0.8)	2.8-6.3	10 672 (5.3)	8.1 (1.1)	5.7-10.2
≥4 d	2602 (1.3)	2.0 (0.7)	0.8-4.2	5501 (2.7)	4.2 (0.9)	1.9-6.4
HW3^f^						
>0 d	8148 (4.0)	6.1 (0.9)	4.2-8.0	5666 (2.8)	4.3 (1.2)	1.4-7.1

Abbreviations: HW, heat wave; MSA, metropolitan statistical area.

^a^ Sum of days in analysis across all MSAs (n = 53) meeting definition.

^b^ Mean annual number of days meeting definition; SD = standard deviation of annual number of days across MSAs.

^c^ Minimum and maximum annual days meeting definition across MSAs (ie, the MSAs with highest and lowest mean days per year).

^d^ Number of days in window exceeding MSA-specific 97.5th percentile temperature threshold.

^e^ Consecutive days in window exceeding location-specific 97.5th percentile temperature threshold.

^f^ Mean temperature (in °C) above the 97.5th percentile during exposure window (eg, 4-day mean temperature − 97.5%; set to 0 if ≤0). The Table shows counts of days with HW3 > 0. Mean (SD) HW3 values among days with HW3 values >0 were 0.80 (0.76) for the 4-day window and 0.68 (0.62) for the 7-day window.

**Figure 2.**
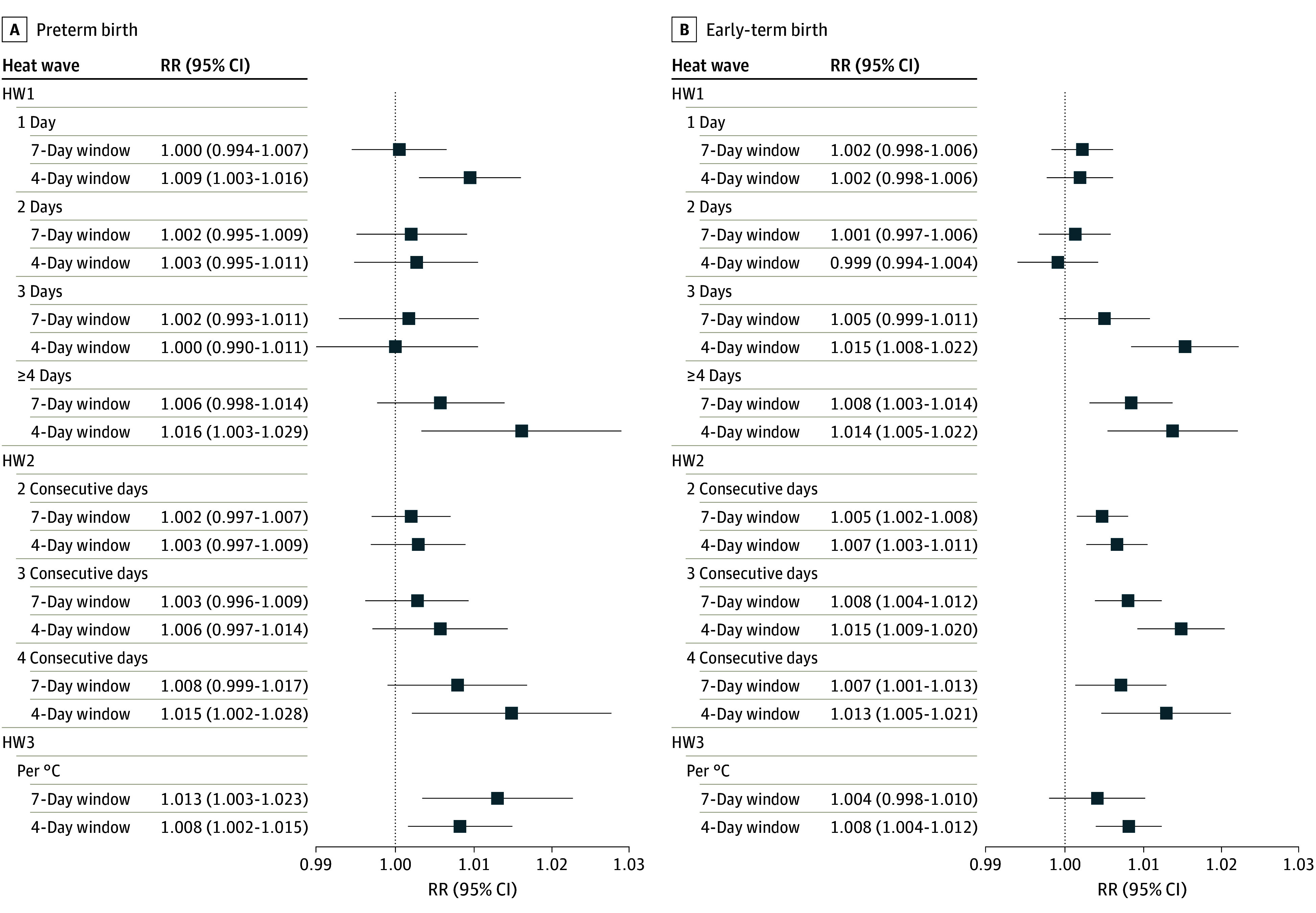
Rate Ratios (RRs) and 95% CIs for Heat Waves Occurring in the 4 Days or 7 Days Preceding Birth (97.5th Percentile Threshold) HW1 indicates heat wave definition 1: total number of hot days in the 4-day (or 7-day) window, represented by an ordinal variable with categories of 0, 1, 2, 3, or 4 days or more; HW2, heat wave definition 2: consecutive hot days in the 4-day (or 7-day) window, represented by binary indicators for 2 or more consecutive days, 3 or more consecutive days, or 4 or more consecutive days; and HW3, heat wave definition 3: mean degrees Celsius over the threshold during the exposure window, a continuous variable calculated as the 4-day (or 7-day) moving mean − the 97.5% threshold, and if less than 0, then set to 0.

Rate ratios and 95% CIs from the primary analysis for all heat wave definitions are shown graphically in Figure 2 and numerically in eTable 3 in Supplement 1. Rate ratios for HW1 are expressed relative to 0 days above the 97.5th percentile threshold in the window, rate ratios for HW2 are relative to days without any 2–consecutive day hot period (or 3-day or 4-day, analyzed in separate models), and HW3 rate ratios are reported per 1 °C increase in mean temperature above the threshold. Heat waves were associated with an increase in daily rates of both preterm and early-term births, with stronger rate ratios observed for more days of heat (HW1), more consecutive days of heat (HW2), and higher mean degrees over the threshold (HW3). After 4 consecutive days of mean temperatures exceeding the local 97.5th percentile (HW2, 4-day), the rate ratio for preterm birth was 1.02 (95% CI, 1.00-1.03), and the rate ratio for early-term birth was 1.01 (95% CI, 1.01-1.02). Each 1 °C increase in mean temperature above the threshold (HW3) was associated with a 1% increase in the rate of both preterm birth (rate ratio, 1.01 [95% CI, 1.00-1.02]) and early-term birth (rate ratio, 1.01 [95% CI, 1.00-1.01]). Rate ratios for the more acute 4-day window tended to be higher than the 7-day window, so sensitivity analyses focus on the 4-day window.

**Figure 3.**
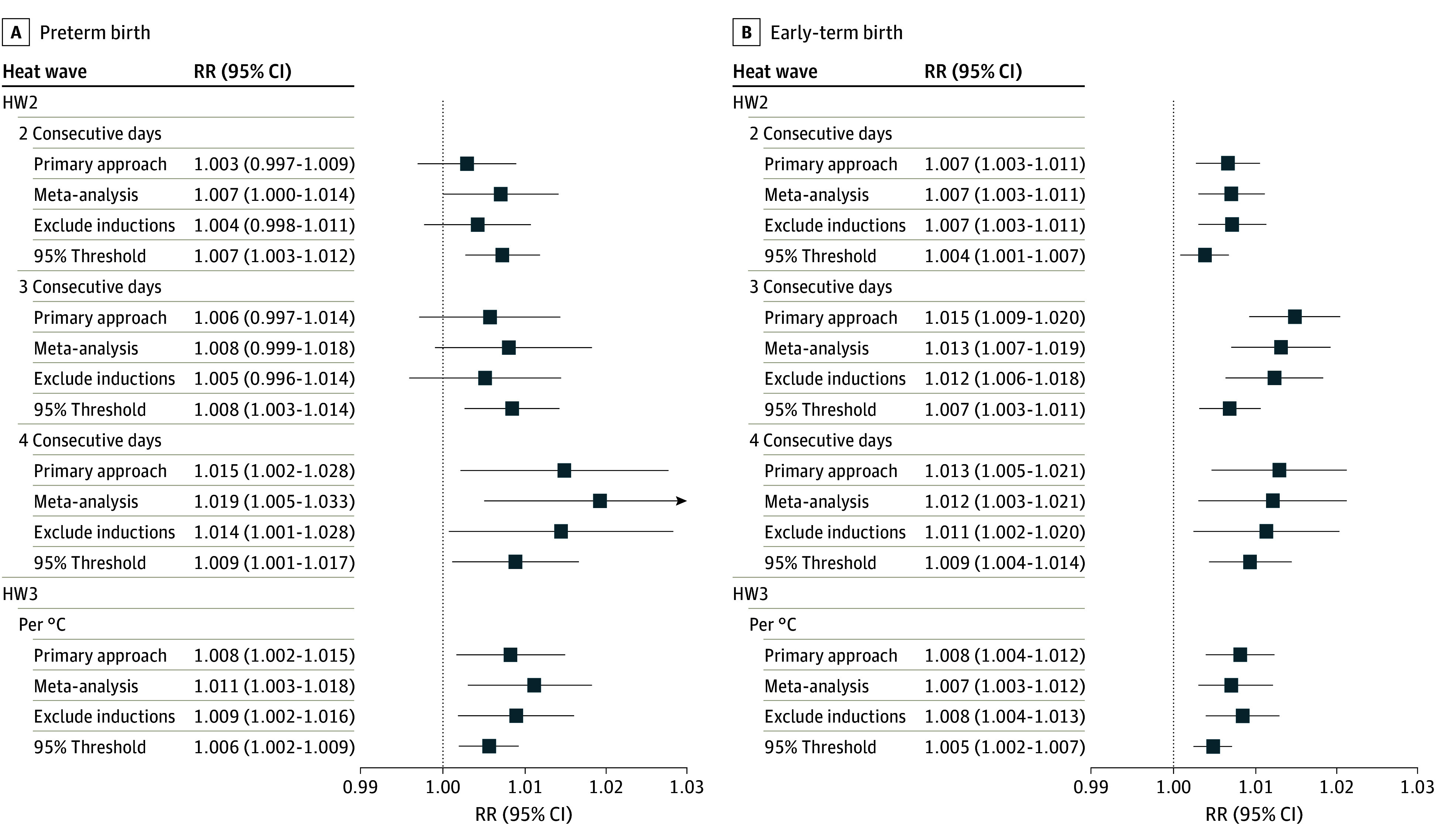
Rate Ratios (RRs) and 95% CIs From Sensitivity Analyses Compared With the Primary Analysis for Heat Waves in the 4-Day Exposure Window HW2 indicates heat wave definition 2: consecutive hot days in the 4-day (or 7-day) window, represented by binary indicators for 2 or more consecutive days, 3 or more consecutive days, or 4 or more consecutive days; HW3, heat wave definition 3: mean degrees Celsius over the threshold during the exposure window, a continuous variable calculated as the 4-day (or 7-day) moving mean − the 97.5% threshold, and if less than 0, then set to 0 (results for heat wave definition 1 are available in eTable 4 in Supplement 1).

Rate ratios and 95% CIs for sensitivity analyses of heat waves (HW2 and HW3) in the 4 days preceding birth are shown in Figure 3 (numerical results, including HW1, are available in eTable 4 in Supplement 1). Use of a 95th percentile temperature threshold instead of 97.5th percentile to define hot days yielded rate ratios that were generally lower than the primary approach, but with narrower 95% CIs due to more days classified as heat waves. Exclusion of induced births (5.9% of preterm births and 7.9% of early-term births), as well as the 53-site random-effects meta-analysis, yielded results highly consistent with the primary approach. When daily maximum or minimum temperature metrics were used to define heat waves, the rate ratios for early-term birth were highest based on minimum temperature, but this pattern was not evident for preterm birth (eFigure 1 in Supplement 1).

**Figure 4.**
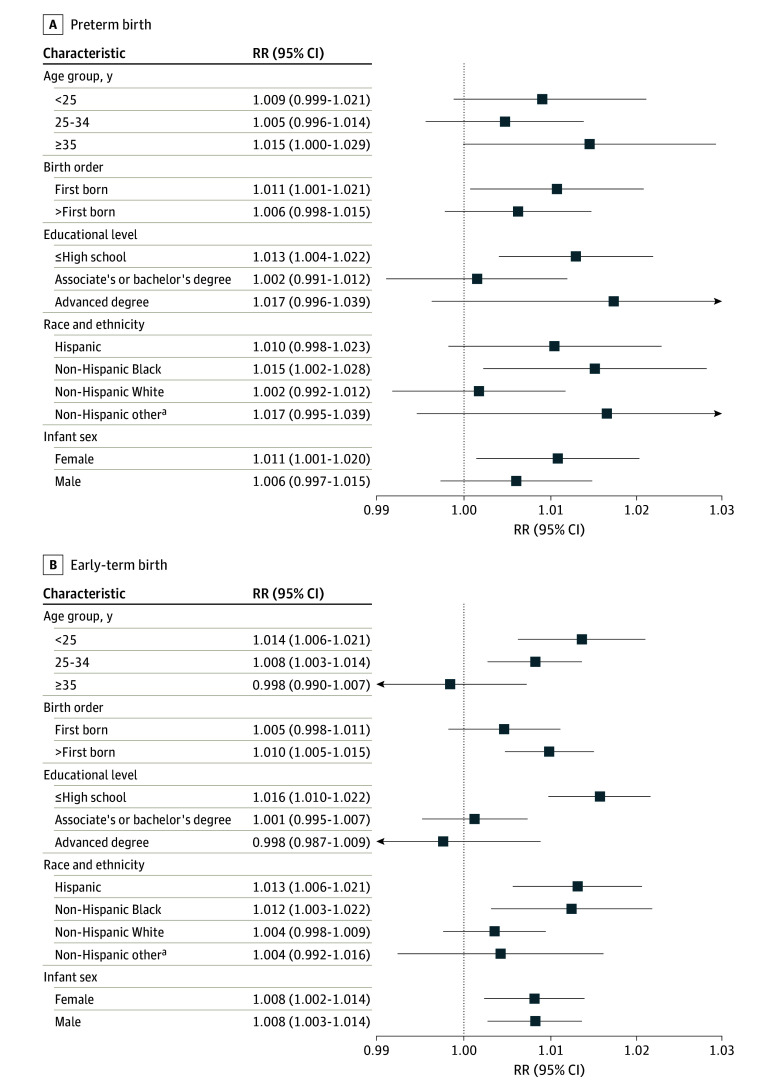
Subgroup Rate Ratios (RRs) and 95% CIs per 1 °C Increase in Mean Temperature Above the 97.5th Percentile Threshold (HW3) During the 4 Days Preceding Birth ^a^Including American Indian, Alaska Native, Asian, Other Pacific Islander, and unknown.

Results of subgroup analyses, stratified by maternal educational level, maternal age, maternal race and ethnicity, infant sex, and live birth order (first, second, third, etc), showed higher heat wave rate ratios among mothers of younger ages, mothers with lower educational levels, and Hispanic or non-Hispanic Black mothers (Figure 4; eFigures 2-6 in Supplement 1). For preterm birth only, rate ratios were elevated among older mothers or those with advanced degrees (subgroups with high overlap). We then analyzed a joint subgroup defined by mothers with multiple susceptibility factors: mothers with high school education or less, from a racial minority group or of Hispanic ethnicity, and age younger than 30 years (instead of <25 years due to small counts). Among this group, the rate ratio for preterm birth was 1.04 (95% CI, 1.02-1.06), and the rate ratio for early-term birth was 1.03 (95% CI, 1.02-1.05) after 4 consecutive hot days (eTable 5 in Supplement 1).

## Discussion

In this nationwide analysis covering 52.8% of US births between 1993 and 2017, we observed a small increase in the rates of preterm and early-term births after multiday periods of locally extreme high temperatures. Increases were more pronounced for heat waves of longer duration and higher temperatures and among population subgroups associated with lower socioeconomic status. We also observed evidence of elevated preterm birth rates, but not early-term rates, after heat waves among mothers 35 years of age or older. Results indicated stronger associations for the shorter 4-day window compared with the 7-day window. To our knowledge, this is the first study to address this study question using national US birth data within the past 2 decades and constitutes the largest study to date on this question.

The magnitude of associations that we observed for preterm birth and heat waves was generally lower than in previous studies, although these studies show considerable heterogeneity.^8,11,12,13,14,15,16,31^ A 2020 six-study meta-analysis yielded a summary odds ratio for preterm birth after heat waves of 1.16 (95% CI, 1.10-1.23) based on populations from Montreal, Quebec, Canada; Alabama; Brisbane, Australia; Rome, Italy; Sabzevar, Iran; and China.^7^ Previous US national analyses have not had access to the birthdate field^32^ or were limited to the 1980s when birthdate and geography were not yet restricted by the NCHS.^9^ Point estimates for preterm birth were similar to those previously reported in the 1980s, although most associations were not statistically significant in the data from the 1980s (the present study included 3.5 times the number of preterm births). We observed stronger associations with preterm birth among certain population subgroups, which is consistent with previous studies that reported stronger associations among the youngest and oldest mothers, members of racial and ethnic minority groups, and mothers with lower educational achievement.^12,33^

Early-term birth has been studied less frequently than preterm birth, but the magnitude of acute increases in early-term birth observed here are consistent with the previous analysis from the 1980s.^9^ Findings are also consistent with a California study (2005-2013 birth records) reporting a less precise hazard ratio of 1.03 (95% CI, 0.98-1.07) for 4 days of temperatures above the 98th percentile in the previous week.^8^ Other smaller studies have reported relative increases of 10% to 30% for prolonged high temperatures in the week preceding early-term birth.^10,15^ Although infants born early term exhibit lower morbidity and mortality relative to preterm births, a small relative increase in the probability of this common outcome (currently >28% of US births^5^) has the potential to affect large numbers of pregnancies.

In the absence of a criterion standard definition of heat wave, we examined several definitions that incorporate duration and temperature intensity. The metrics were selected a priori and were informed by previous analyses of a broader suite of heat metrics (eg, including apparent temperature and excessive heat factor).^9^ We chose not to examine apparent temperature, a measure incorporating relative humidity, because estimates from the 1980s were similar between those defined using temperature vs apparent temperature, and results from other studies making within-location temporal contrasts indicated little difference when humidity was considered.^8,34^ Because all contrasts in our study were temporal (ie, comparing event rates over time within an MSA population), humidity differences between cities would not contribute to the estimated associations. Heat waves defined using mean, minimum, or maximum daily temperature thresholds were correlated and yielded mostly similar results. We observed some evidence for a stronger association between minimum temperature heat wave metrics and early-term birth, but not for preterm birth (eFigure 1 in Supplement 1), and 95% CIs overlapped.

### Strengths and Limitations

This study has some strengths. Several design and analysis choices limited the potential for confounding in this study. Because all contrasts were temporal, potential confounders were limited to those that vary day to day. One particular a priori concern was the seasonal pattern of conception in the US that led to a peak of pregnancies in July.^26,28^ A previously conducted simulation study evaluated several designs and statistical model specifications under these confounding conditions to inform our approach.^29^ Based on these simulations, which indicated a small positive bias in time-stratified case-crossover models, we selected the time-series approach as implemented by Vicedo-Cabrera et al^30^ to account for the changing gestational age distribution of the risk set. Long-term time trends were controlled by adjusting for birth year, and the smooth function on day of season adjusted for any recurrent seasonal trends over the course of the warm season. Weekday was independent of the exposure (heat waves) but was included because it reduced overdispersion.

A major strength of this study is the multisite approach and the application of consistent, validated^28^ methods across locations. The primary analyses modeled all data together, controlling for MSA with fixed effects, but concern about residual confounding by MSA-specific seasonal or long-term time trends motivated the meta-analysis that allowed all covariate associations to vary by MSA (year, day of season, and weekday). Results of this random-effects meta-analysis strengthened our confidence in the primary analysis results.

This study also has some limitations. Observed rate ratios in this study represent the net population-level changes in rates of the outcome after heat waves. They cannot isolate the association of physical exposure to heat waves with the outcome because some pregnant individuals undoubtedly modify behavior in response to heat. Some of these behavioral modifications, such as avoiding physical exertion, may actually decrease the rates of preterm and early-term births.^35^ The population-level estimated effects also obscure subpopulation vulnerabilities.^33^ Variables systematically collected on birth records are only crude proxies of social and biologic factors that might modify the risks of heat waves, such as access to housing with reliable air conditioning, or coexposures, such as stress and underlying health conditions that increase susceptibility to the outcomes. In addition, our study excluded births in rural areas, where mothers could experience more vulnerability to extreme heat due to less access to cooling.

## Conclusions

This 25-year cohort study based on 53 million births provides compelling population-based evidence of increased preterm and early-term birth rates in response to heat waves. The modest but robust elevated associations were strongest in the 4 days preceding birth and for longer durations of heat and higher temperatures. The findings also add to evidence that the effects of extreme heat events are not distributed evenly among population subgroups.

## Notes

Supplement 1.
eMethods.
**eTable 1.** Federal Information Processing System (FIPS) Codes for 416 Counties Included in the 50 Largest U.S. Metropolitan Statistical Areas in the 2010 Census **eTable 2.** Total Births in Each MSA (or Sub-MSA for Los Angeles and New York City) Over the Study Period 1993-2017 and Counts of Preterm (28-37 Weeks) and Early-Term Births (37-38 Weeks) Included in Analysis **eTable 3.** Adjusted Rate Ratios for Preterm and Early-Term Birth for Heatwaves Occurring in the 4-Day or 7-Days Before Birth, Shown Graphically in Figure 2 **eTable 4.** Adjusted Rate Ratios for Preterm and Early-Term Birth for Heatwaves Occurring in the 4-Day Before Birth (Lag 0-3) From Sensitivity Analyses: (1) Defining Heat Waves Based on a 95% Temperature Threshold (2) Excluding Medically-Induced Deliveries From Preterm and Early-Term Daily Counts (97.5%ile Threshold) (3) Random Effects Meta-Analysis of MSA-Specific Rate Ratios (97.5%ile Threshold) **eFigure 1.** Rate Ratios and 95% Confidence Intervals for Heatwaves Based on 97.5%ile Thresholds of Minimum, Mean, and Maximum Temperature for the 4-Day Exposure Window **eFigure 2.** Rate Ratios and 95% Confidence Intervals for Heatwaves in the 4 Days Preceeding Birth and Preterm and Early Term Birth, Stratified by Maternal Race/Ethnicity **eFigure 3.** Rate Ratios and 95% Confidence Intervals for Heatwaves in the 4 Days Preceeding Birth and Preterm and Early Term Birth, Stratified by Maternal Education Level **eFigure 4.** Rate Ratios and 95% Confidence Intervals for Heatwaves Occurring in the 4 Days Preceeding Birth and Preterm and Early Term Birth, Stratified by Maternal Age Group **eFigure 5.** Rate Ratios and 95% Confidence Intervals for Heatwaves in the 4 Days Preceeding Birth and Preterm and Early Term Birth, Stratified by Infant Sex **eFigure 6.** Rate Ratios and 95% Confidence Intervals for Heatwaves in the 4 Days Preceeding Birth and Preterm and Early Term Birth, Stratified by Live Birth Order **eTable 5.** Adjusted Rate Ratios and 95% Confidence Intervals for Heatwaves in the 4 Days Preceeding Birth and Preterm and Early-Term Birth, Among Mothers ≤29 Years of Age and ≤ a High School Education and a Race or Ethnicity Other Than non-Hispanic White

Supplement 2.
Data Sharing Statement
