# Supplementary material for: Preterm and Early-Term Delivery After Heat Waves in 50 US Metropolitan Areas
Source: JAMA Netw Open. 2024 May 24;7(5):e2412055. doi: 10.1001/jamanetworkopen.2024.12055 (PMC11127119; doi:10.1001/jamanetworkopen.2024.12055)
Supplement: Supplement 1. — eMethods. eTable 1. Federal Information Processing System (FIPS) Codes for 416 Counties Included in the 50 Largest U.S. Metropolitan Statistical Areas in the 2010 Census eTable 2. Total Births in Each MSA (or Sub-MSA for Los Angeles and New York City) Over the Study Period 1993-2017 and Counts of Preterm (28-37 Weeks) and Early-Term Births (37-38 Weeks) Included in Analysis eTable 3. Adjusted Rate Ratios for Preterm and Early-Term Birth for Heatwaves Occurring in the 4-Day or 7-Days Before Birth, Shown Graphically in Figure 2 eTable 4. Adjusted Rate Ratios for Preterm and Early-Term Birth for Heatwaves Occurring in the 4-Day Before Birth (Lag 0-3) From Sensitivity Analyses: (1) Defining Heat Waves Based on a 95% Temperature Threshold (2) Excluding Medically-Induced Deliveries From Preterm and Early-Term Daily Counts (97.5%ile Threshold) (3) Random Effects Meta-Analysis of MSA-Specific Rate Ratios (97.5%ile Threshold) eFigure 1. Rate Ratios and 95% Confidence Intervals for Heatwaves Based on 97.5%ile Thresholds of Minimum, Mean, and Maximum Temperature for the 4-Day Exposure Window eFigure 2. Rate Ratios and 95% Confidence Intervals for Heatwaves in the 4 Days Preceeding Birth and Preterm and Early Term Birth, Stratified by Maternal Race/Ethnicity eFigure 3. Rate Ratios and 95% Confidence Intervals for Heatwaves in the 4 Days Preceeding Birth and Preterm and Early Term Birth, Stratified by Maternal Education Level eFigure 4. Rate Ratios and 95% Confidence Intervals for Heatwaves Occurring in the 4 Days Preceeding Birth and Preterm and Early Term Birth, Stratified by Maternal Age Group eFigure 5. Rate Ratios and 95% Confidence Intervals for Heatwaves in the 4 Days Preceeding Birth and Preterm and Early Term Birth, Stratified by Infant Sex eFigure 6. Rate Ratios and 95% Confidence Intervals for Heatwaves in the 4 Days Preceeding Birth and Preterm and Early Term Birth, Stratified by Live Birth Order eTable 5. Adjusted Rate Ratios and 95% Confidence Intervals for Heatwav [file jamanetwopen-e2412055-s001.pdf]

Supplementary Online Content

Darrow LA, Huang M, Warren JL, et al. Preterm and early-term delivery after heat waves in 50 US metropolitan areas. *JAMA Netw Open.* 2024;7(5):e2412055. doi:10.1001/jamanetworkopen.2024.12055

eMethods.
eTable 1. Federal Information Processing System (FIPS) Codes for 416 Counties Included in the 50 Largest U.S. Metropolitan Statistical Areas in the 2010 Census
eTable 2. Total Births in Each MSA (or Sub-MSA for Los Angeles and New York City) Over the Study Period 1993-2017 and Counts of Preterm (28-37 Weeks) and Early-Term Births (37-38 Weeks) Included in Analysis
eTable 3. Adjusted Rate Ratios for Preterm and Early-Term Birth for Heatwaves Occurring in the 4-Day or 7-Days Before Birth, Shown Graphically in Figure 2
eTable 4. Adjusted Rate Ratios for Preterm and Early-Term Birth for Heatwaves Occurring in the 4-Day Before Birth (Lag 0-3) From Sensitivity Analyses: (1) Defining Heat Waves Based on a 95% Temperature Threshold (2) Excluding Medically-Induced Deliveries from Preterm and Early-Term Daily Counts (97.5%ile Threshold) (3) Random Effects Meta-Analysis of MSA-Specific Rate Ratios (97.5%ile Threshold)
eFigure 1. Rate Ratios and 95% Confidence Intervals for Heatwaves Based on 97.5%ile Thresholds of Minimum, Mean, and Maximum Temperature for the 4-Day Exposure Window
eFigure 2. Rate Ratios and 95% Confidence Intervals for Heatwaves in the 4 Days Preceeding Birth and Preterm and Early-Term Birth, Stratified by Maternal Race/Ethnicity
eFigure 3. Rate Ratios and 95% Confidence Intervals for Heatwaves in the 4 Days Preceeding Birth and Preterm and Early-Term Birth, Stratified by Maternal Education Level
eFigure 4. Rate Ratios and 95% Confidence Intervals for Heatwaves Occurring in the 4 Days Preceeding Birth and Preterm and Early Term Birth, Stratified by Maternal Age Group
eFigure 5. Rate Ratios and 95% Confidence Intervals for Heatwaves in the 4 Days Preceeding Birth and Preterm and Early Term Birth, Stratified by Infant Sex
eFigure 6. Rate Ratios and 95% Confidence Intervals for Heatwaves in the 4 Days Preceeding Birth and Preterm and Early Term Birth, Stratified by Live Birth Order
eTable 5. Adjusted Rate Ratios and 95% Confidence Intervals for Heatwaves in the 4 Days Preceeding Birth and Preterm and Early-Term Birth, Among Mothers ≤29 Years of Age and ≤ a High School Education and a Race or Ethnicity Other Than non-Hispanic White

© 2024 Darrow LA et al. *JAMA Network Open.*

This supplementary material has been provided by the authors to give readers additional information about their work.

© 2024 Darrow LA et al. *JAMA Network Open.*

eMethods.

Statistical Models.

Poisson regression models included an offset term for the pregnancies-at-risk and their gestational age distribution (Vicedo-Cabrera et al., 2014). The general model is:

$$Y_i|\lambda_i \sim \text{Poisson}(\lambda_i),$$

$$\ln(\lambda_i) = \ln\left(z_i^{(w)}\right) + \beta_0 + \beta_1 * Heat\ wave_i$$

where Y_i is the outcome count on study day *i*, and the corresponding offset $z_i^{(w)}$ for study day *i* is

$$z_i^{(w)} = \sum_{j=28}^{36}(Z_{ij} * W_j)$$

where Z_{ij} is the number of pregnancies-at-risk on study day *i* and gestational week *j* (*j*=28, 29,…, 36 weeks for preterm, or 37,38 for early term) and W_j is the probability of giving birth at gestational age *j*, calculated from the empirical birth data in each MSA. Older gestational ages have greater of probability of birth, and therefore greater contribution to $z_i^{(w)}$. Hence we can conceptualize $z_i^{(w)}$ as the expected count of preterm (or early-term) births on a given day based on the pregnancies-at-risk and their stage of gestation. By including this offset in the model we control for the imbalances in expected outcome counts across days

The primary models for **Heatwave definition 1** (HW1) took the form:

$$\ln(\lambda_{im}) = \ln\left(z_{im}^{(w)}\right) + \beta_0 + \sum_{h=1}^{4}(\beta_h * HW1_{him}) + \sum_{m=1}^{52}(\beta_m * MSA_m) + \sum_{j=1}^{6}(\beta_j * weekday_{ji})$$

$$+ \sum_{k=1}^{24}(\beta_k * year_{ki}) + \sum_{d=1}^{4}(\beta_d * DOS_{di})$$

where Y_{im} is the count of preterm births on day *i* in MSA *m*; $z_{im}^{(w)}$ is the offset described above on day *i* in MSA *m*; $HW1_h$ is the categorical variable HW1 representing the number of days in the exposure window (4 or 7 days) exceeding the temperature threshold (categories are 0,1,2,3,4+, reference=0) in day *i* in MSA *m*; MSA_m are the indicators for MSA (or sub-MSA for New York City and Los Angeles); $weekday_{ji}$ are indicators for day of week for day *i*; $year_{ki}$ are indicators for birth year for day *i*; and DOS_{di} is the cubic spline on day of warm season (1-153) with one knot on July 15.

Heatwave definition 2 (HW2) replaces the categorical HW1 in the model above with a binary indicator for presence of ≥2-consecutive hot days in the window (or ≥3-consecutive or ≥4-consecutive days, run in separate models) on day *i* in MSA *m*. $(\beta_1 * HW2_{im})$

Heatwave definition 3 (HW3) replaces the categorical HW1 in the model above with a continuous term for the average degrees above the threshold during the window (set to 0 if average temperature was below the threshold, i.e., most days, see Table 1) on day *i* in MSA *m*; Values above zero increased with temperature and duration of heat in the window. $(\beta_1 * HW3_{im})$

© 2024 Darrow LA et al. *JAMA Network Open.*

MSA-specific analyses involved 53 separate models for each heatwave definition and were specified the same as the model above except for removal of MSA. Because analyses were stratified by MSA, all other covariate regression parameters (weekday, year, day of season) were also estimated separately by MSA.

<u>Meta-analysis.</u>

We propose a multivariate random effects meta-analysis model for the vector of MSA-specific regression parameter estimates ($\widehat{\boldsymbol{\beta}}_i$) and corresponding variance/covariance matrices ($\hat{\Sigma}_i$). The model assumes that each estimate has an independent multivariate normal (MVN) distribution with mean vector equal to the true, but unobserved vector of parameters ($\boldsymbol{\beta}_i$) and variance/covariance defined by $\hat{\Sigma}_i$. Then, $\boldsymbol{\beta}_i$ is assumed to follow a MVN distribution with mean vector equal to the pooled effects ($\boldsymbol{\mu}$) and variance/covariance defined by Ω. The model is given as

$$\widehat{\boldsymbol{\beta}}_i \sim \text{MVN}\left(\boldsymbol{\beta}_i, \hat{\Sigma}_i\right), i = 1, \dots, 53$$

$$\boldsymbol{\beta}_i \sim \text{MVN}(\boldsymbol{\mu}, \Omega).$$

We work in the Bayesian setting for model fitting and specify the following weakly informative prior distributions for the model parameters:

- $\boldsymbol{\mu}^{\text{T}} = \left(\mu_1, \dots, \mu_p\right)$; $\mu_j \sim \text{N}(0{,}100^2)$; *p* is the number of estimates in the vector;
- $\Omega^{-1} \sim \text{Wishart}\left(p + 1, I_p\right)$

We fit all models using Markov chain Monte Carlo sampling techniques and collect 10,000 samples from the joint point posterior distribution of interest, after discarding the first 10,000 during an initialization phase and thinning the remaining 100,000 by a factor of 10 to reduce posterior autocorrelation. Convergence was assessed by visually inspecting the trace plot for each model parameter. We report posterior summaries for $\exp\left\{\mu_j\right\}$ (i.e., rate ratio scale) including posterior means and 95% quantile-based equal-tailed credible intervals. In the situation where the MSA-specific estimate is a scalar (i.e. *p*=1), and not a vector, we use the same model structure where variance/covariance matrices are replaced by scalar variance parameters, and the Wishart distribution is replaced by a Uniform(0,100) for the standard deviation parameter.

© 2024 Darrow LA et al. *JAMA Network Open.*

eTable 1. Federal Information Processing System (FIPS) Codes for 416 Counties Included in the 50 Largest U.S. Metropolitan Statistical Areas in the 2010 Census

Metropolitan Statistical Area	FIPS code [state code (first two) and county code (last 3)]
Atlanta-Sandy Springs-Marietta, GA	13013, 13015, 13035, 13045, 13057, 13063, 13067, 13077, 13085, 13089, 13097, 13113, 13117, 13121, 13135, 13143, 13149, 13151, 13159, 13171, 13199, 13217, 13223, 13227, 13231, 13247, 13255, 13297
Austin-Round Rock-San Marcos, TX	48021, 48055, 48209, 48453, 48491
Baltimore-Towson, MD	24003, 24005, 24013, 24025, 24027, 24035, 24510
Birmingham-Hoover, AL	01007, 01009, 01021, 01073, 01115, 01117, 01127
Boston-Cambridge-Quincy, MA-NH	25009, 25017, 25021, 25023, 25025, 33015, 33017
Buffalo-Niagara Falls, NY	36029, 36063
Charlotte-Gastonia-Rock Hill, NC-SC	37007, 37025, 37071, 37119, 37179, 45091
Chicago-Joliet-Naperville, IL-IN-WI	17031, 17037, 17043, 17063, 17089, 17093, 17097, 17111, 17197, 18073, 18089, 18111, 18127, 55059
Cincinnati-Middletown, OH-KY-IN	18029, 18047, 18115, 21015, 21023, 21037, 21077, 21081, 21117, 21191, 39015, 39017, 39025, 39061, 39165
Cleveland-Elyria-Mentor, OH	39035, 39055, 39085, 39093, 39103
Columbus, OH	39041, 39045, 39049, 39089, 39097, 39117, 39129, 39159
Dallas-Fort Worth-Arlington, TX	48085, 48113, 48119, 48121, 48139, 48231, 48251, 48257, 48367, 48397, 48439, 48497
Denver-Aurora-Broomfield, CO	08001, 08005, 08014, 08019, 08031, 08035, 08039, 08047, 08059, 08093
Detroit-Warren-Livonia, MI	26087, 26093, 26099, 26125, 26147, 26163
Hartford-West Hartford-East Hartford, CT	09003, 09007, 09013
Houston-Sugar Land-Baytown, TX	48015, 48039, 48071, 48157, 48167, 48201, 48291, 48339, 48407, 48473
Indianapolis-Carmel, IN	18011, 18013, 18057, 18059, 18063, 18081, 18097, 18109, 18133, 18145
Jacksonville, FL	12003, 12019, 12031, 12089, 12109
Kansas City, MO-KS	20059, 20091, 20103, 20107, 20121, 20209, 29013, 29025, 29037, 29047, 29049, 29095, 29107, 29165, 29177
Las Vegas-Paradise, NV	32003
Los Angeles-Long Beach-Santa Ana[a]	Los Angeles County: 06037<br>Orange County: 06059
Louisville/Jefferson County, KY-IN	18019, 18043, 18061, 18175, 21029, 21103, 21111, 21163, 21179, 21185, 21211, 21215, 21223
Memphis, TN-MS-AR	05035, 28033, 28093, 28137, 28143, 47047, 47157, 47167
Miami-Fort Lauderdale-Pompano Beach, FL	12011, 12086, 12099
Milwaukee-Waukesha-West Allis, WI	55079, 55089, 55131, 55133
Minneapolis-St. Paul-Bloomington, MN-WI	27003, 27019, 27025, 27037, 27053, 27059, 27123, 27139, 27141, 27163, 27171, 55093, 55109

© 2024 Darrow LA et al. *JAMA Network Open.*

New York-Northern New Jersey-Long Island[b]	New York City: 36005, 36047, 36061, 36081, 36085<br>Long Island: 36059, 36103<br>New Jersey/Other New York: 34003, 34013, 34017, 34019, 34023, 34025, 34027, 34029, 34031, 34035, 34037, 34039, 36079, 36087, 36119, 42103
Nashville-Davidson--Murfreesboro--Franklin, TN	47015, 47021, 47037, 47043, 47081, 47111, 47147, 47149, 47159, 47165, 47169, 47187, 47189
New Orleans-Metairie-Kenner, LA	22051, 22071, 22075, 22087, 22089, 22095, 22103
Oklahoma City, OK	40017, 40027, 40051, 40081, 40083, 40087, 40109
Orlando-Kissimmee-Sanford, FL	12069, 12095, 12097, 12117
Philadelphia-Camden-Wilmington, PA-NJ-DE-MD	10003, 24015, 34005, 34007, 34015, 34033, 42017, 42029, 42045, 42091, 42101
Phoenix-Mesa-Glendale, AZ	04013, 04021
Pittsburgh, PA	42003, 42005, 42007, 42019, 42051, 42125, 42129
Portland-Vancouver-Hillsboro, OR-WA	41005, 41009, 41051, 41067, 41071, 53011, 53059
Providence-New Bedford-Fall River, RI-MA	25005, 44001, 44003, 44005, 44007, 44009
Raleigh-Cary, NC	37069, 37101, 37183
Richmond, VA	51007, 51033, 51036, 51041, 51049, 51053, 51075, 51085, 51087, 51097, 51101, 51109, 51127, 51145, 51149, 51183, 51570, 51670, 51730, 51760
Riverside-San Bernardino-Ontario, CA	06065, 06071
Sacramento--Arden-Arcade--Roseville, CA	06017, 06061, 06067, 06113
Salt Lake City, UT	49035, 49043, 49045
San Antonio-New Braunfels, TX	48013, 48019, 48029, 48091, 48187, 48259, 48325, 48493
San Diego-Carlsbad-San Marcos, CA	06073
San Francisco-Oakland-Fremont, CA	06001, 06013, 06041, 06075, 06081
San Jose-Sunnyvale-Santa Clara, CA	06069, 06085
Seattle-Tacoma-Bellevue, WA	53033, 53053, 53061
St. Louis, MO-IL	17005, 17013, 17027, 17083, 17117, 17119, 17133, 17163, 29055, 29071, 29099, 29113, 29183, 29189, 29219, 29221, 29510
Tampa-St. Petersburg-Clearwater, FL	12053, 12057, 12101, 12103
Virginia Beach-Norfolk-Newport News, VA-NC	37053, 51073, 51093, 51095, 51115, 51181, 51199, 51550, 51650, 51700, 51710, 51735, 51740, 51800, 51810, 51830
Washington-Arlington-Alexandria, DC-VA-MD-WV	11001, 24009, 24017, 24021, 24031, 24033, 51013, 51043, 51059, 51061, 51107, 51153, 51177, 51179

[a] Subdivided for analysis into two locations due to size: Los Angeles County and Orange County
[b] Subdivided for analysis into three locations due to size: New York City, Long Island, New Jersey/Other NY Counties

© 2024 Darrow LA et al. *JAMA Network Open.*

eTable 2. Total Births in Each MSA (or Sub-MSA for Los Angeles and New York City) Over the Study Period 1993-2017 and Counts of Preterm (28-37 Weeks) and Early-Term Births (37-38 Weeks) Included in Analysis[a]

Metropolitan Statistical Area	Births 1993-2017	Births (%)	Early-term births[a]	Preterm births[a]
Overall	55,748,869	100%	5,795,313	2,153,609
Atlanta-Sandy Springs-Marietta, GA	1,778,666	3.2	200,125	72,419
Austin-Round Rock-San Marcos, TX	569,235	1.0	62,125	21,147
Baltimore-Towson, MD	861,155	1.5	86,207	36,623
Birmingham-Hoover, AL	370,357	0.7	39,694	18,060
Boston-Cambridge-Quincy, MA-NH	1,374,657	2.5	130,815	41,938
Buffalo-Niagara Falls, NY	327,448	0.6	31,223	12,136
Charlotte-Gastonia-Rock Hill, NC-SC	562,322	1.0	55,011	22,555
Chicago-Joliet-Naperville, IL-IN-WI	3,354,287	6.0	339,351	136,508
Cincinnati-Middletown, OH-KY-IN	714,808	1.3	71,734	27,676
Cleveland-Elyria-Mentor, OH	654,848	1.2	67,068	26,955
Columbus, OH	630,275	1.1	64,991	25,677
Dallas-Fort Worth-Arlington, TX	2,334,571	4.2	262,892	94,117
Denver-Aurora-Broomfield, CO	855,407	1.5	90,254	32,171
Detroit-Warren-Livonia, MI	1,425,358	2.6	142,712	59,569
Hartford-W. Hartford-E. Hartford, CT	337,423	0.6	33,620	10,561
Houston-Sugar Land-Baytown, TX	2,218,337	4.0	257,557	98,049
Indianapolis-Carmel, IN	612,677	1.1	67,270	24,430
Jacksonville, FL	431,873	0.8	45,308	18,789
Kansas City, MO-KS	695,369	1.2	70,667	25,095
Las Vegas-Paradise, NV	604,852	1.1	69,671	28,671
Los Angeles 1 (Los Angeles County)	3,718,654	6.7	387,533	130,420
Los Angeles 2 (Orange County)	1,082,911	1.9	112,246	31,514
Louisville/Jefferson County, KY-IN	403,148	0.7	45,204	18,383
Memphis, TN-MS-AR	483,184	0.9	52,981	24,781
Miami-Ft Lauderdale-Pompano Beach, FL	1,697,134	3.0	196,755	77,948
Milwaukee-Waukesha-West Allis, WI	524,912	0.9	52,387	20,942
Minneapolis-St. Paul-Bloomington, MN-WI	1,109,237	2.0	103,804	34,923
Nashville-Davidson--Murfreesboro--Franklin, TN	512,443	0.9	60,020	21,806
New Orleans-Metairie-Kenner, LA	433,567	0.8	48,241	21,679
New York 1 (New York City)	3,005,892	5.4	294,231	120,990
New York 2 (Long Island)	853,303	1.5	87,146	29,866
New York 3 (New Jersey/NY Other)	2,531,157	4.5	256,245	94,509
Oklahoma City, OK	434,871	0.8	49,090	18,480
Orlando-Kissimmee-Sanford, FL	634,134	1.1	73,381	29,989
Philadelphia-Camden-Wilmington, PA-NJ-DE-MD	1,885,400	3.4	180,682	72,175
Phoenix-Mesa-Glendale, AZ	1,446,483	2.6	161,355	60,933

© 2024 Darrow LA et al. *JAMA Network Open.*

**Metropolitan Statistical Area**	Births 1993-2017	Births (%)	Early-term births[a]	Preterm births[a]
Portland-Vancouver-Hillsboro, OR-WA	689,632	1.2	66,623	21,466
Providence-New Bedford-Fall River, RI-MA	460,671	0.8	42,569	15,746
Raleigh-Cary, NC	347,350	0.6	31,367	13,078
Richmond, VA	379,807	0.7	44,334	17,067
Riverside-San Bernardino-Ontario, CA	1,482,457	2.7	155,003	52,405
Sacramento--Arden-Arcade--Roseville, CA	677,021	1.2	61,725	20,166
Salt Lake City, UT	478,095	0.9	55,978	17,507
San Antonio-New Braunfels, TX	752,180	1.3	92,952	35,690
San Diego-Carlsbad-San Marcos, CA	1,122,082	2.0	105,614	34,825
San Francisco-Oakland-Fremont, CA	1,358,408	2.4	125,568	39,920
San Jose-Sunnyvale-Santa Clara, CA	662,205	1.2	63,616	18,571
Seattle-Tacoma-Bellevue, WA	1,076,095	1.9	98,085	33,967
St. Louis, MO-IL	907,677	1.6	97,661	38,676
Tampa-St. Petersburg-Clearwater, FL	761,944	1.4	81,529	31,799
Virginia Beach-Norfolk-Newport News, VA-NC	588,177	1.1	59,935	23,372
Washington-Arlington-Alexandria, DC-VA-MD-WV	1,899,196	3.4	202,213	73,385

[a] analyzed counts of early-term birth (37-38 weeks gestation) and preterm birth (28-36 weeks gestation) restricted to warm season (May-September) singletons with nonmissing gestational age.

© 2024 Darrow LA et al. *JAMA Network Open.*

eTable 3. Adjusted[a] Rate Ratios for Preterm and Early-Term Birth for Heatwaves Occurring in the 4-Day or 7-Days Before Birth, Shown Graphically in Figure 2

	Preterm birth				Early-term birth			
	4-day window		7-day window		4-day window		7-day window	
	RR(95%CI)	p	RR(95%CI)	p	RR(95%CI)	p	RR(95%CI)	p
**HW1 (total hot days, reference=0 days)**								
1	1.009 (1.003, 1.016)	0.008	1.000 (0.994, 1.007)	0.701	1.002 (0.998, 1.006)	<0.0001	1.002 (0.998, 1.006)	0.015
2	1.003 (0.995, 1.011)		1.002 (0.995, 1.009)		0.999 (0.994, 1.004)		1.001 (0.997, 1.006)	
3	1.000 (0.990, 1.011)		1.002 (0.993, 1.011)		1.015 (1.008, 1.022)		1.005 (0.999, 1.011)	
4+	1.016 (1.003, 1.029)		1.006 (0.998, 1.014)		1.014 (1.005, 1.022)		1.008 (1.003, 1.014)	
**HW2 (consecutive hot days, yes vs. no)[b]**								
2 consecutive	1.003 (0.997, 1.009)	0.347	1.002 (0.997, 1.007)	0.432	1.007 (1.003, 1.011)	0.001	1.005 (1.001, 1.008)	0.004
3 consecutive	1.006 (0.997, 1.014)	0.120	1.003 (0.996, 1.009)	0.409	1.015 (1.009, 1.020)	<0.0001	1.008 (1.004, 1.012)	0.0002
4 consecutive	1.015 (1.002, 1.028)	0.023	1.008 (0.999, 1.017)	0.083	1.013 (1.005, 1.021)	0.0023	1.007 (1.001, 1.013)	0.016
**HW3 (per degree above threshold, continuous)**								
per 1 °C	1.008 (1.002, 1.015)	0.014	1.013 (1.003, 1.023)	0.008	1.008 (1.004, 1.012)	0.0002	1.004 (0.998, 1.010)	0.192

[a] Models are adjusted for MSA, birth year, weekday, and day of season, scale factors from the Poisson models were 1.06 for preterm and 1.13 for early-term.
[b] Indicators for 2, 3 or 4-consecutive day metrics analyzed in separate models

© 2024 Darrow LA et al. *JAMA Network Open.*

eTable 4. Adjusted[a] Rate Ratios for Preterm and Early-Term Birth for Heatwaves Occurring in the 4-Day Before Birth (Lag 0-3) From Sensitivity Analyses: (1) Defining Heat Waves Based on a 95% Temperature Threshold (2) Excluding Medically-Induced Deliveries from Preterm and Early-Term Daily Counts (97.5%ile Threshold) (3) Random Effects Meta-Analysis of MSA-Specific Rate Ratios (97.5%ile Threshold). Results shown graphically in Figure 3 (HW2, HW3); numerical results for primary analysis shown in eTable 3.

	**Preterm birth**			**Early-term birth**		
	**95% threshold**	**Exclude inductions**	**Meta-analysis**	**95% threshold**	**Exclude inductions**	**Meta-analysis**
**HW1 (total hot days, reference=0 days)**						
1	1.004 (0.999, 1.009)	1.013 (1.007, 1.020)	1.012 (1.005, 1.019)	1.005 (1.001, 1.008)	1.003 (0.998, 1.007)	1.003 (0.998, 1.008)
2	1.007 (1.001, 1.014)	1.006 (0.998, 1.015)	1.007 (0.999, 1.015)	1.002 (0.998, 1.006)	1.002 (0.997, 1.008)	1.002 (0.997, 1.007)
3	1.010 (1.003, 1.018)	1.000 (0.989, 1.012)	1.005 (0.994, 1.016)	1.003 (0.998, 1.008)	1.013 (1.006, 1.020)	1.013 (1.006, 1.020)
4+	1.011 (1.003, 1.019)	1.016 (1.003, 1.030)	1.022 (1.008, 1.036)	1.011 (1.005, 1.016)	1.012 (1.003, 1.021)	1.013 (1.005, 1.022)
**HW2 (consecutive hot days, yes vs. no)[b]**						
2 consecutive	1.007 (1.003, 1.012)	1.004 (0.998, 1.011)	1.007 (1.000, 1.014)	1.004 (1.001, 1.007)	1.007 (1.003, 1.011)	1.007 (1.003, 1.011)
3 consecutive	1.008 (1.003, 1.014)	1.005 (0.996, 1.014)	1.008 (0.999, 1.018)	1.007 (1.003, 1.011)	1.012 (1.006, 1.018)	1.013 (1.007, 1.020)
4 consecutive	1.009 (1.001, 1.017)	1.014 (1.001, 1.028)	1.019 (1.004, 1.034)	1.009 (1.004, 1.014)	1.011 (1.002, 1.020)	1.012 (1.003, 1.021)
**HW3 (per degree above threshold, continuous)**						
per 1 °C	1.006 (1.002, 1.009)	1.009 (1.002, 1.016)	1.011 (1.003, 1.018)	1.005 (1.002, 1.007)	1.008 (1.004, 1.013)	1.007 (1.003, 1.012)

[a] Models are adjusted for MSA, birth year, weekday, and day of season
[b] Indicators for 2, 3 or 4-consecutive day metrics analyzed in separate models

© 2024 Darrow LA et al. *JAMA Network Open.*

eFigure 1. Rate Ratios and 95% Confidence Intervals for Heatwaves Based on 97.5%ile Thresholds of Minimum, Mean, and Maximum Temperature for the 4-Day Exposure Window. Mean temperature estimates are the same as those shown in Figure 2 (primary analysis).

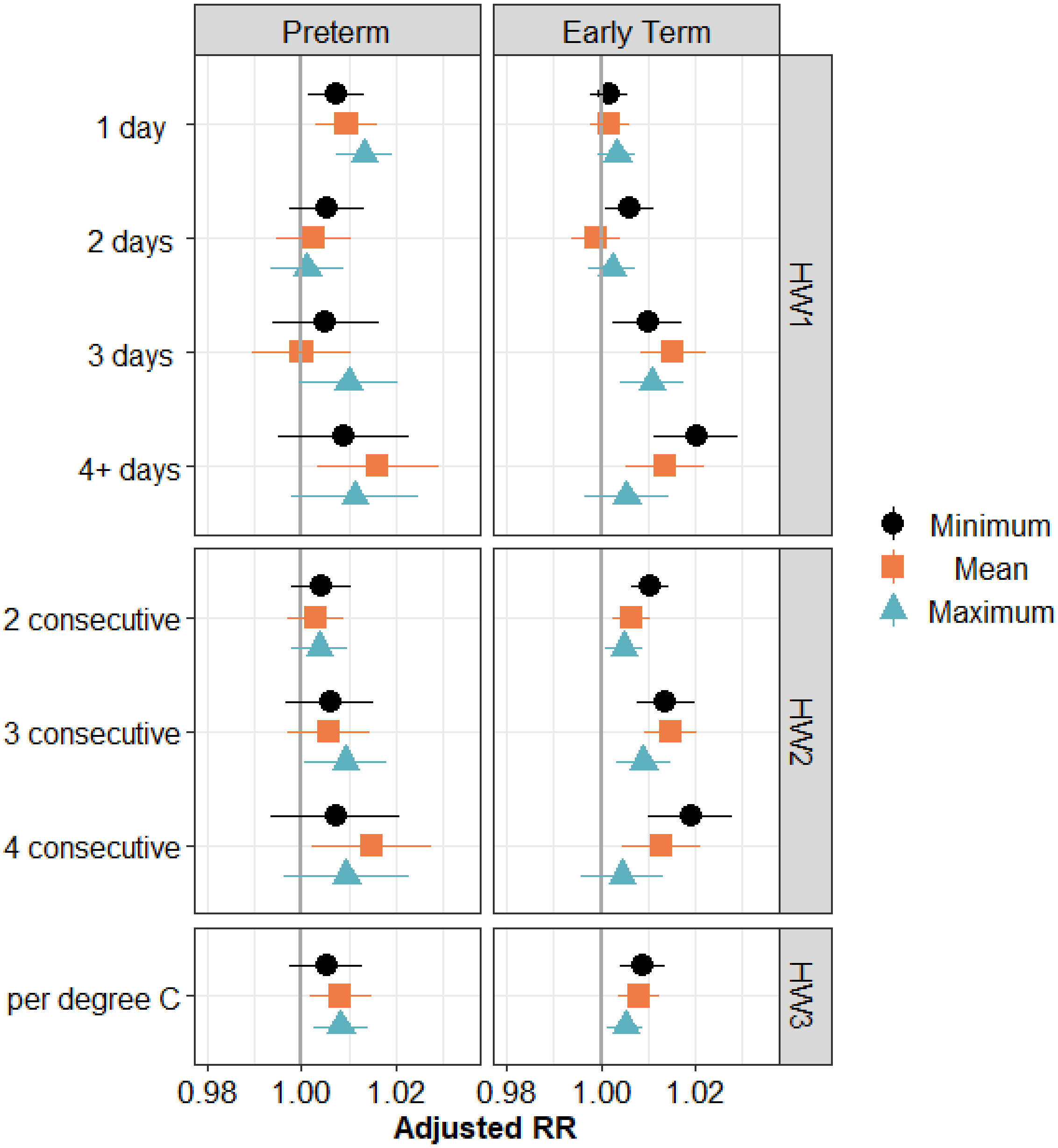

© 2024 Darrow LA et al. *JAMA Network Open.*

eFigure 2. Rate Ratios and 95% Confidence Intervals for Heatwaves in the 4 Days Preceeding Birth and Preterm and Early Term Birth, Stratified by **Maternal Race/Ethnicity**

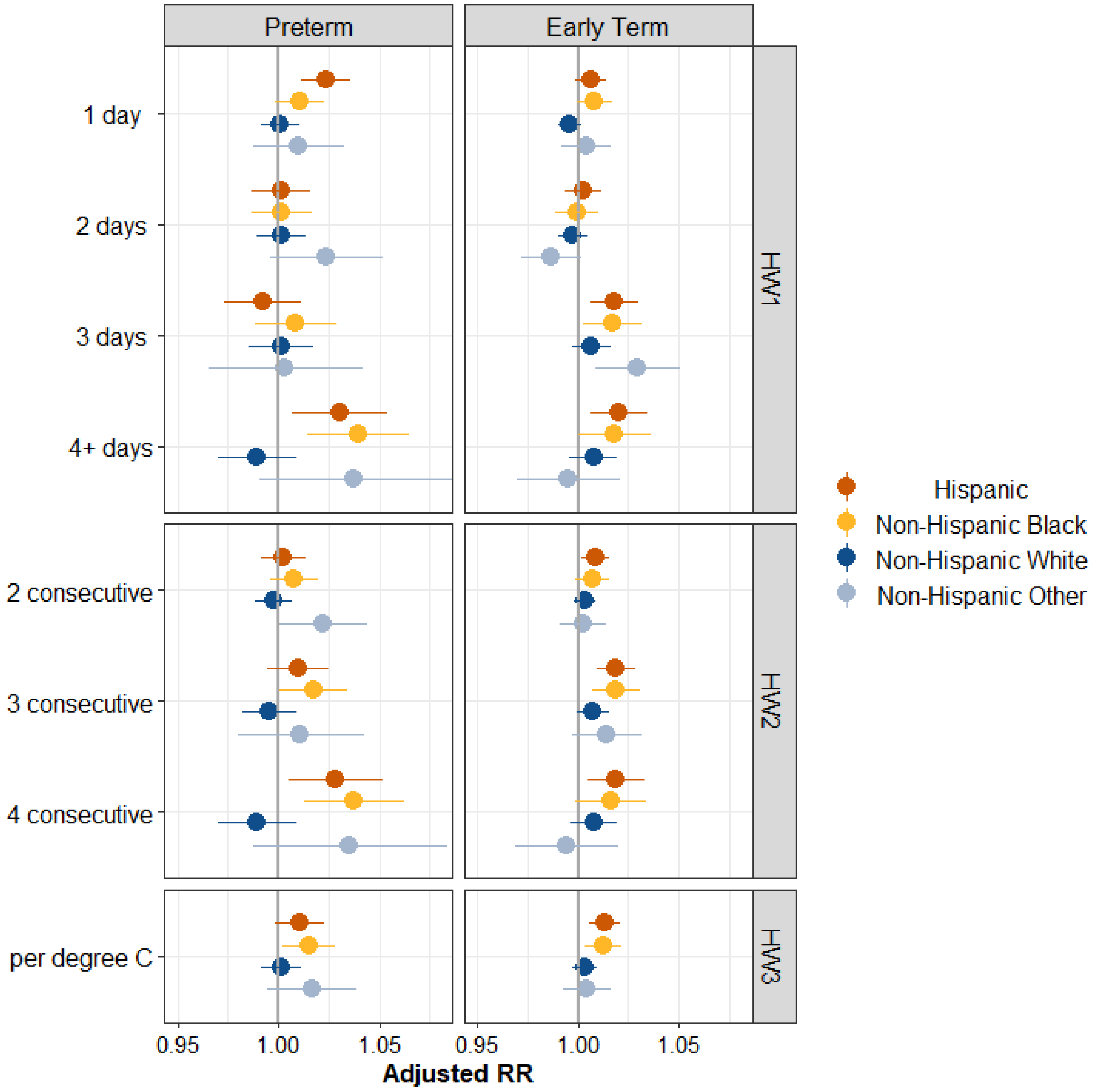

© 2024 Darrow LA et al. *JAMA Network Open.*

eFigure 3. Rate Ratios and 95% Confidence Intervals for Heatwaves in the 4 Days Preceeding Birth and Preterm and Early Term Birth, Stratified by **Maternal Education Level.**

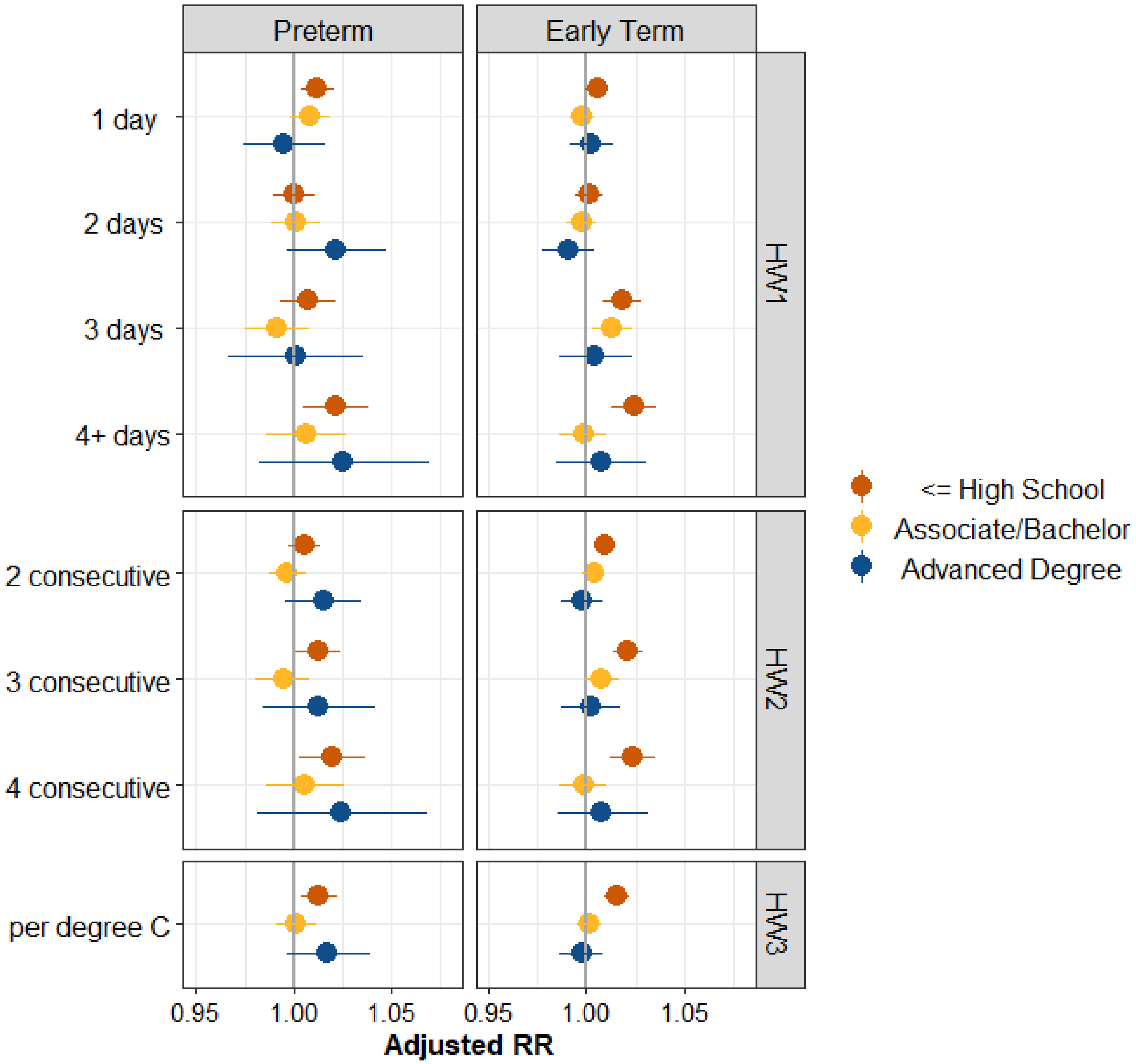

© 2024 Darrow LA et al. *JAMA Network Open.*

eFigure 4. Rate Ratios and 95% Confidence Intervals for Heatwaves Occurring in the 4 Days Preceeding Birth and Preterm and Early Term Birth, Stratified by **Maternal Age Group.**

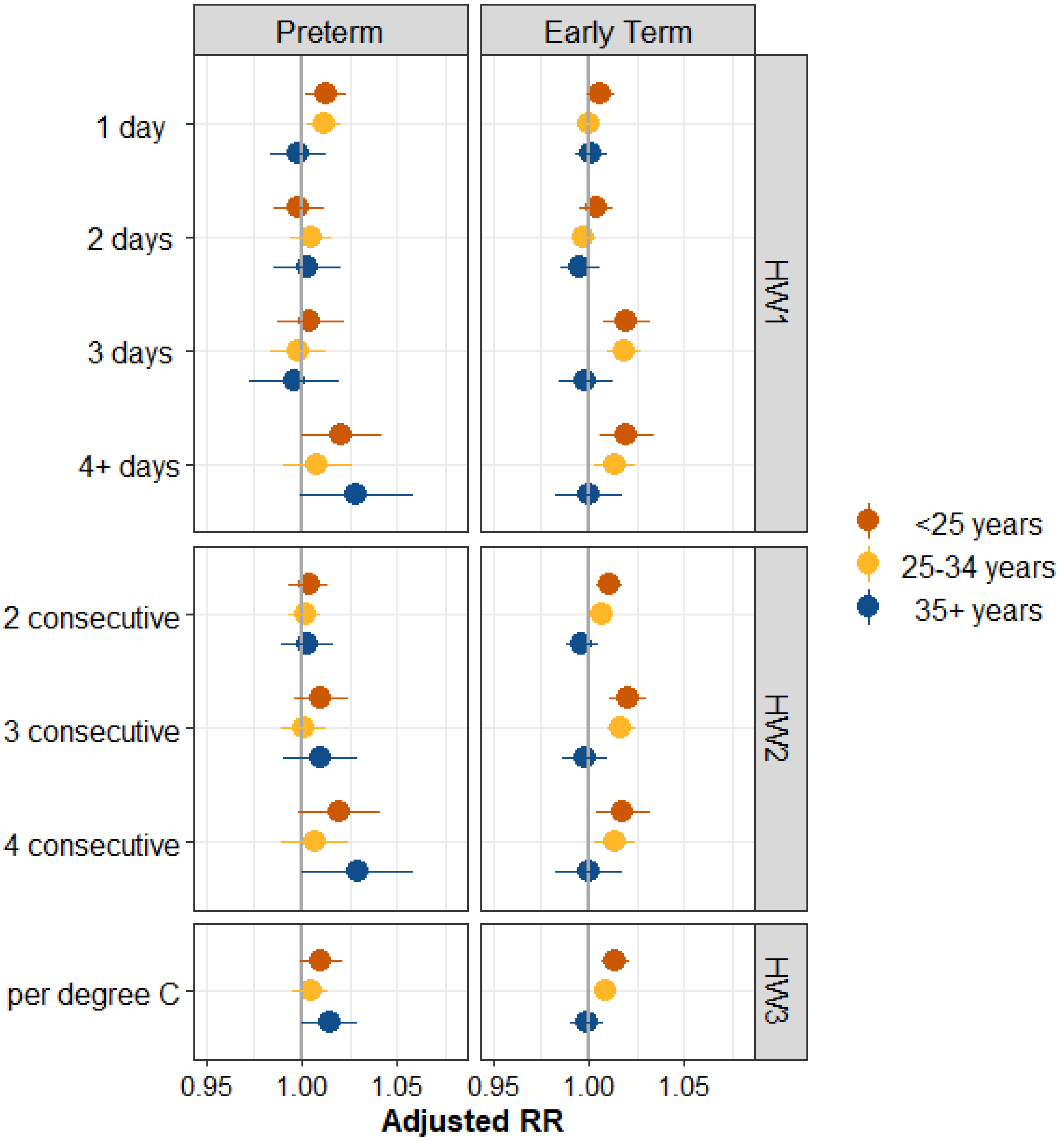

© 2024 Darrow LA et al. *JAMA Network Open.*

eFigure 5. Rate Ratios and 95% Confidence Intervals for Heatwaves in the 4 Days Preceeding Birth and Preterm and Early Term Birth, Stratified by **Infant Sex**.

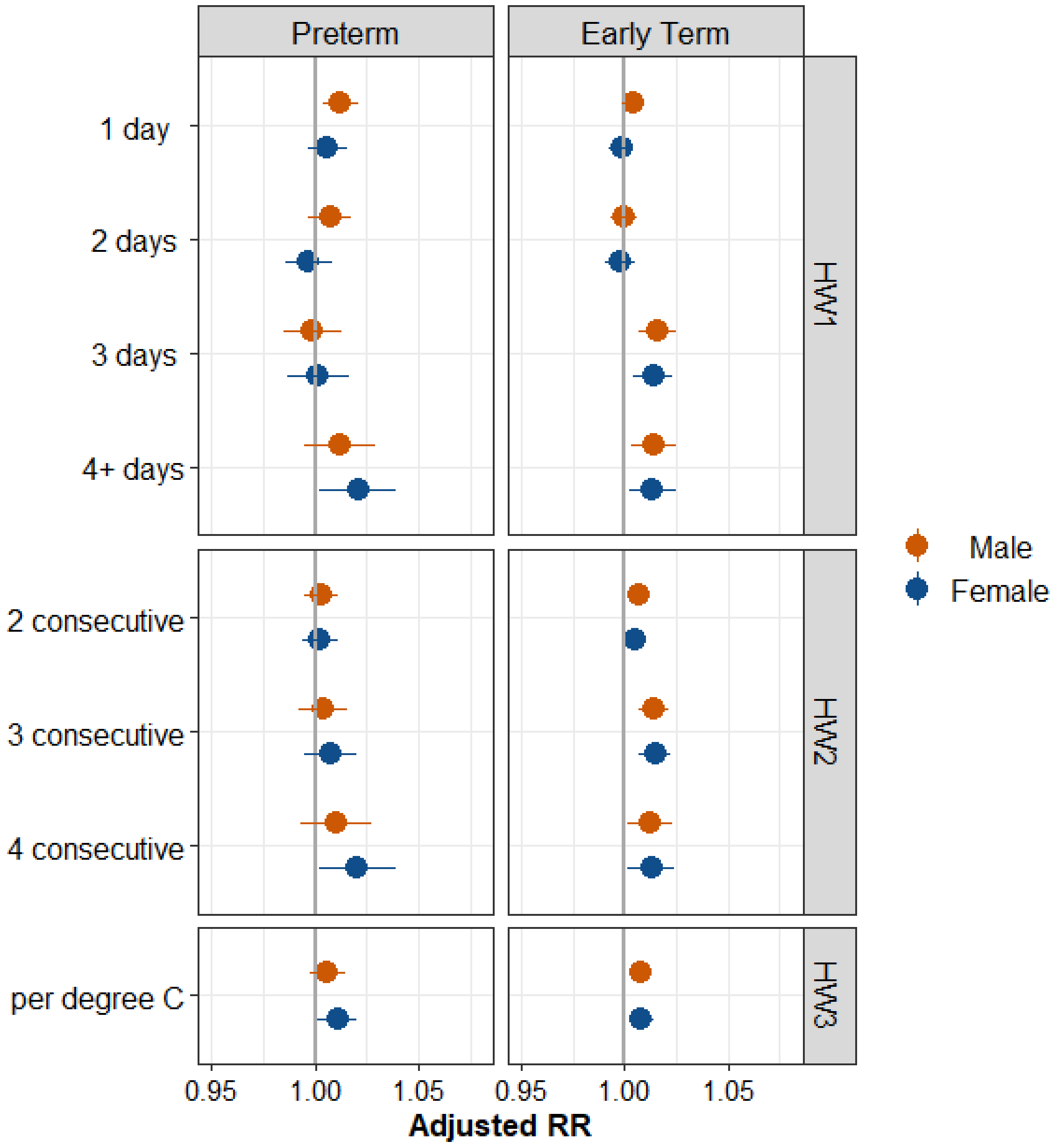

© 2024 Darrow LA et al. *JAMA Network Open.*

eFigure 6. Rate Ratios and 95% Confidence Intervals for Heatwaves in the 4 Days Preceeding Birth and Preterm and Early Term Birth, Stratified by **Live Birth Order.**

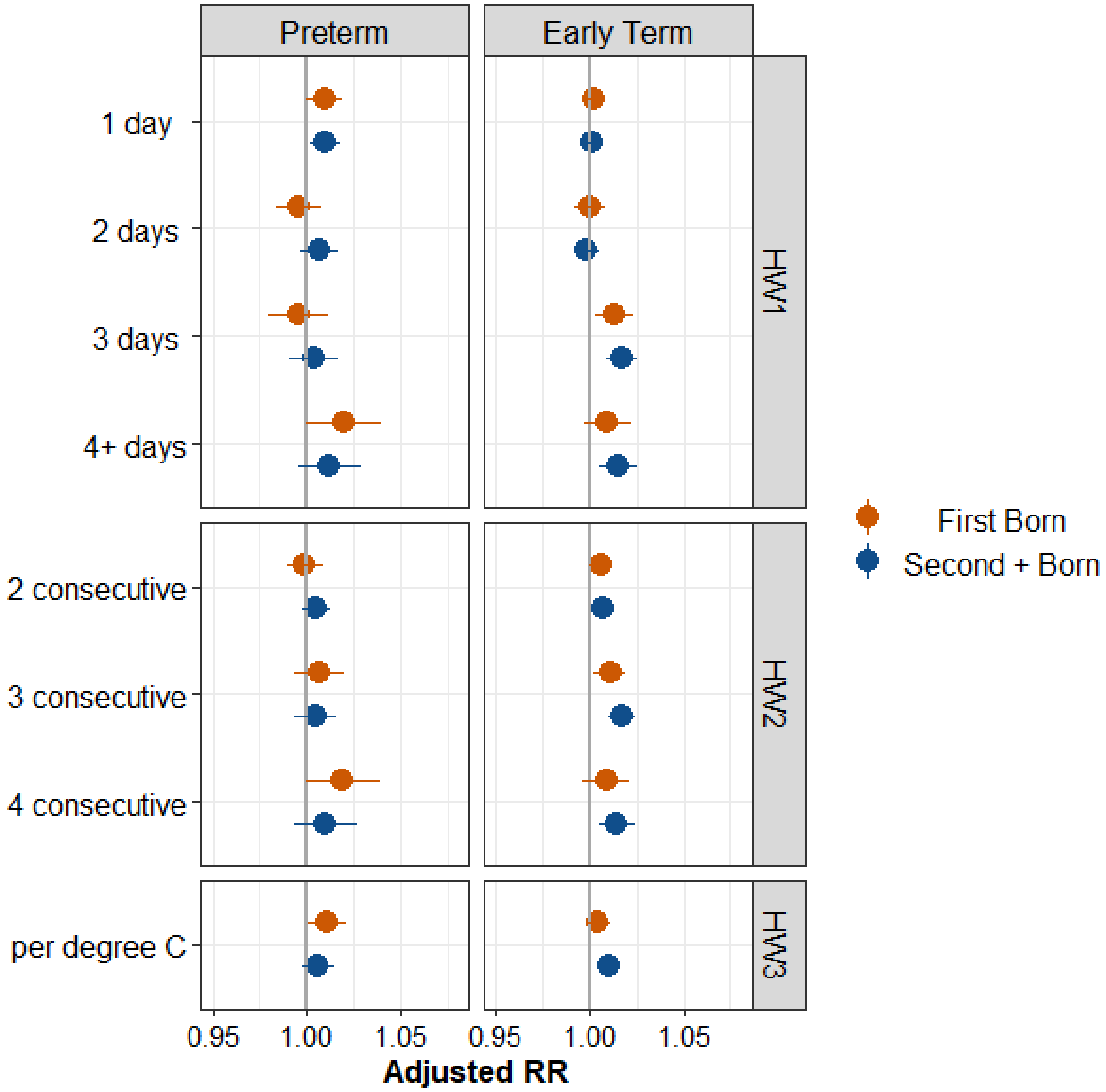

© 2024 Darrow LA et al. *JAMA Network Open*.

eTable 5. Adjusted Rate Ratios and 95% Confidence Intervals for Heatwaves in the 4 Days Preceeding Birth and Preterm and Early-Term Birth, Among Mothers ≤29 Years of Age and ≤ a High School Education and a Race or Ethnicity Other Than non-Hispanic White.

	Preterm Birth	Early-term Birth
**HW1 (total hot days, reference=0 days)**		
1	1.020 (1.008, 1.032)	1.004 (0.996, 1.012)
2	1.001 (0.986, 1.015)	1.008 (0.998, 1.018)
3	0.997 (0.978, 1.017)	1.025 (1.012, 1.039)
4+	1.043 (1.020, 1.068)	1.035 (1.019, 1.051)
**HW2 (consecutive hot days, yes vs. no)**[b]		
2 consecutive	1.008 (0.997, 1.019)	1.017 (1.010, 1.025)
3 consecutive	1.015 (0.999, 1.031)	1.029 (1.018, 1.040)
4 consecutive	1.041 (1.018, 1.065)	1.032 (1.016, 1.048)
**HW3 (per degree above threshold, continuous)**		
per 1 °C	1.015 (1.003, 1.028)	1.023 (1.014, 1.031)

© 2024 Darrow LA et al. *JAMA Network Open.*
