# Supplementary material for: Preterm and Early-Term Delivery After Heat Waves in 50 US Metropolitan Areas
Source: JAMA Netw Open. 2024 May 24;7(5):e2412055. doi: 10.1001/jamanetworkopen.2024.12055 (PMC11127119; doi:10.1001/jamanetworkopen.2024.12055)
Supplement: Supplement 2. — Data Sharing Statement [file jamanetwopen-e2412055-s002.pdf]

# Data Sharing Statement

Darrow. Preterm and Early-Term Delivery After Heat Waves in 50 US Metropolitan Areas. *JAMA Netw Open*. Published May 24, 2024. doi:10.1001/jamanetworkopen.2024.12055

**Data**
**Data available:** No

**Additional Information**
**Explanation for why data not available:** The birth datasets and restricted variables utilized in this study are accessible only onsite at the National Center for Health Statistics (CDC) after an approval process and data usage fee https://www.cdc.gov/nchs/data_access/vitalstatsonline.htm). DAYMET meteorological data are publicly available for download (https://daymet.ornl.gov/overview).
